# Cloning and Characterization of a Human Genomic Sequence that Alleviates Repeat-Induced Gene Silencing

**DOI:** 10.1371/journal.pone.0153338

**Published:** 2016-04-14

**Authors:** Miki Fukuma, Yuto Ganmyo, Osamu Miura, Takashi Ohyama, Noriaki Shimizu

**Affiliations:** 1 Graduate School of Biosphere Science, Hiroshima University, Higashi-hiroshima, Hiroshima, Japan; 2 Graduate School of Advanced Science and Engineering, Waseda University, Shinjuku-ku, Tokyo, Japan; Cleveland Clinic Foundation, UNITED STATES

## Abstract

Plasmids bearing a mammalian replication initiation region (IR) and a nuclear matrix attachment region (MAR) are spontaneously amplified in transfected mammalian cells, and such amplification generates chromosomal homogeneously staining regions (HSRs) or extrachromosomal double minutes (DMs). This method provides a novel, efficient, and rapid way to establish cells that stably produce high levels of recombinant proteins. However, because IR/MAR plasmids are amplified as repeats, they are frequently targeted by repeat-induced gene silencing (RIGS), which silences a variety of repeated sequences in transgenes and the genome. To address this problem, we developed a novel screening system using the IR/MAR plasmid to isolate human genome sequences that alleviate RIGS. The screen identified a 3,271 bp sequence (B-3-31) that elevated transgene expression without affecting the amplification process. Neither non-B structure (i.e., the inverted repeats or bending) nor known epigenetic modifier elements such as MARs, insulators, UCOEs, or STARs could explain the anti-silencing activity of B-3-31. Instead, the activity was distributed throughout the entire B-3-31 sequence, which was extremely A/T-rich and CpG-poor. Because B-3-31 effectively and reproducibly alleviated RIGS of repeated genes, it could be used to increase recombinant protein production.

## Introduction

Amplification of oncogenes plays a pivotal role in carcinogenesis (reviewed in [1,2]). Amplified genes localize either to extrachromosomal double minutes (DMs) or chromosomal homogeneously staining regions (HSRs). We previously showed that a plasmid bearing both a mammalian replication initiation region (IR) and a nuclear matrix attachment region (MAR) was spontaneously amplified in transfected mammalian cells to hundreds to thousands of copies per cell, and that these amplifications generated DMs or HSRs [3,4]. Because this process likely mimics the episomal model of gene amplification [5] in cultured cells, this method has been used to investigate the mechanism of gene amplification [6–8].

Both the IR and MAR sequences are necessary for amplification [9]. In the IRs from the dihydrofolate reductase (*DHFR*), c-*myc*, and ß-*globin* loci, the minimal sequences required for amplification contain multiple elements necessary for replication initiation [9,10]. At least during the initial stages after transfection, an IR/MAR plasmid is maintained extrachromosomally. During that time, the plasmid multimerizes to form a large circular molecule in which the plasmid sequences are arranged as a direct repeat [3]. If multimerization proceeds extensively, the plasmid multimer itself appears as a DM [6]. On the other hand, if the multimer is integrated into the chromosome arm, it initiates the breakage-fusion-bridge (BFB) cycle, generating a large and homogenous HSR in human COLO 320 cells or small fine ladder—type HSR in hamster CHO DG44 cells [6]. Such HSRs can be further lengthened via activation of chromosomal fragile sites by methotrexate (Mtx) treatment [8,11].

Efficient production of recombinant proteins, such as biopharmaceuticals, in animal cells is an important technology that still requires additional improvements (reviewed in [12]). IR/MAR gene amplification provides an excellent platform for swiftly and efficiently producing recombinant proteins. The superiority of this approach relative to previously established systems was demonstrated for expression of human recombinant cyclooxygenase-1 in human HEK 293T cells [13] and humanized recombinant antibody in CHO DG44 cells [11,14]. In the latter case, IR/MAR gene amplification rapidly generated a cell line that could secrete antibody at a rate of 29.4 pg/cell/day [14] or 45.1 pg/cell/day (Araki et al., unpublished data) in serum-free suspension culture.

One drawback of the IR/MAR method is that the amplified and repeated sequences frequently suffer from gene silencing. This problem is commonly observed when various methods are employed to increase transgene copy number. If this problem could be overcome, it would increase the chance of obtaining clonal cells that express higher levels of recombinant protein during long term culture. The most severe silencing is observed in long homogeneous HSRs consisting of the plasmid repeat in COLO 320 cells. Such repeats are replicated at the last stage of S phase of the cell cycle [15], and only a few loci within such HSRs are transcriptionally active [16]. Moreover, chromatin within these HSRs contains histone H3K9 trimethylation, a marker of heterochromatin [16]. The fine ladder—type HSRs observed in CHO DG44 cells are superior in this respect, but they may also be silenced to some extent, depending on the clone and the culture period [8,11].

Repeated genes are often silenced by repeat-induced gene silencing (RIGS), a mechanism that involves RNA interference (reviewed in [17]). RIGS plays a roles in transposon inactivation and the establishment of constitutive heterochromatin at the centromere and telomere. Because amplified arrays created by the IR/MAR approach are tandem plasmid repeats [3], and because RIGS silences tandem repeats of transfected genes [18], it is likely that RIGS is also responsible for silencing of amplified arrays of IR/MAR plasmid sequences.

In this study, we extensively screened a human genome library constructed using the IR/MAR vector and identified a human genomic sequence (B-3-31) that alleviates the silencing of amplified plasmid arrays. We analyzed the sequence of B-3-31 to elucidate the determinants responsible for its anti-silencing activity and reveal its mechanism of action.

## Materials and Methods

### Construction of primary *E*. *coli* library

**Fig 1:**
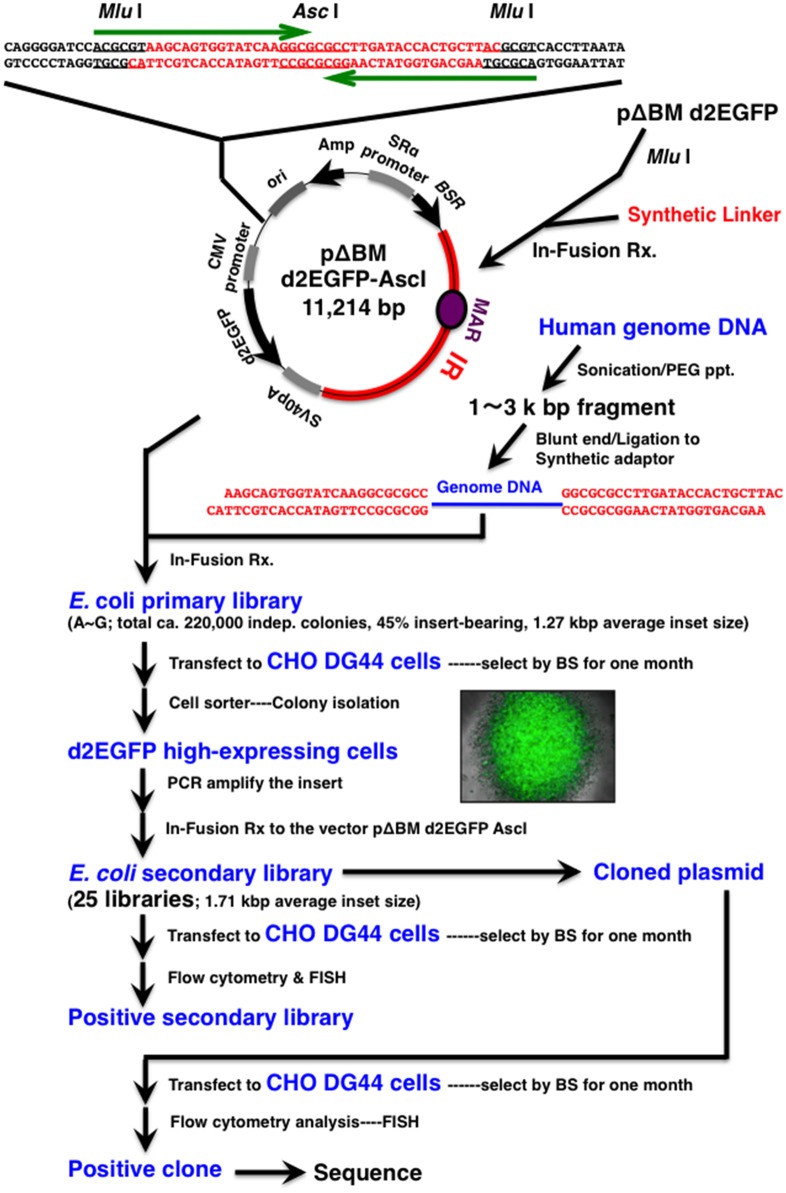
Flowchart of library construction and screening. **The** *E*. *coli* primary library was constructed by using a human genomic DNA fragment and the pΔBM-d2EGFP AscI vector plasmid, which contains IR/MAR, *BSR*, and *d2EGFP* expression cassettes. DNA from the library was transfected into CHO DG44 cells, and stable transformants showing high d2EGFP expression were isolated. The human genomic inserts were recovered from the cells and were used to construct the secondary *E*. *coli* library using the same IR/MAR vector (pΔBM-d2EGFP AscI). The library DNA was again transfected into CHO DG44 cells, and a secondary library showing high d2EGFP expression was identified. The plasmids were cloned from the secondary library and transfected into CHO DG44 cells. The cloned plasmid that showed higher d2EGFP expression than that of the vector plasmid was identified. The human genomic insert in this plasmid was sequenced. For details, see Methods.

The construction of pΔBM-d2EGFP was described previously [19]. This plasmid contains a 4.6 kb IR sequence from a non-coding region of the *DHFR* locus, including a sequence that exhibits *in vitro* MAR activity [4]. It also harbors the *d2EGFP* gene, which encodes an enhanced green fluorescence protein with a short intracellular half-life (2 hours), and the blasticidine resistance gene (*BSR*). A double-stranded synthetic oligonucleotide was inserted into the *Mlu*I site of pΔBM-d2EGFP (Fig 1, upper most line) using the In-Fusion HD Cloning Kit w/Cloning Enhancer (Clontech). The resultant plasmid (pΔBM-d2EGFP-AscI), whose structure is shown in Fig 1, contains an *Asc*I cloning site suitable for insertion of a human genomic fragment. The *Asc*I site is located at the center of a 2 × 25 base palindrome, and insertion at this site abolishes the palindromic structure.

Genomic DNA was obtained from COLO 320DM (ATCC CCL 220) human colorectal carcinoma cell line using a standard protocol. The DNA was sheared by sonication under conditions that preferentially produce 1,000–3,000 bp fragments. Fragments longer than 600 bp were precipitated by addition of 6.7% polyethylene glycol (PEG) 8000 (Promega) and 10 mM Mg_2_Cl_2_ (final concentrations). The recovered DNA fragments were blunted using KOD DNA polymerase (Toyobo), followed by blunt-end ligation with a synthetic oligonucleotide adaptor whose sequence was identical to the sequence flanking the *Asc*I site of the vector (pΔBM-d2EGFP-AscI), highlighted in red in Fig 1. The ligated DNA was purified by PEG precipitation as described above and inserted into the *Asc*I site of pΔBM-d2EGFP-AscI using the In-Fusion HD Cloning Kit (Clontech). The product was directly transfected into *E*. *coli* DH5α competent cells and plated on LB-Agar containing 100 μg/ml ampicillin. The human genome insert was directly amplified from *E*. *coli* colonies by PCR using a single primer (In-Fusion AscI-S; 5’-AAGCAGTGGTATCAA-3’). *E*. *coli* colonies from agar plates were suspended in LB medium, and the DNA was purified using the PureLink HiPure Plasmid Midiprep Kit (Life Technologies) without further growth in liquid medium.

### Transfection to CHO cells and screening

Purified plasmid DNA from the primary library was transfected into CHO DG44 cells using Lipofectamine 2000 (Life Technologies). CHO DG44 cells were cultured in Ham’s F-12 medium supplemented with 10% FCS as described [14]. Transfectants were selected with 10 μg/ml blasticidin S hydrochloride (BS; Funakishi) starting 2 days after transfection. Multiple colonies appeared 2–3 weeks after transfection, and these cells were detached and sub-cultured. After the stable transfectants began to grow logarithmically, the concentration of BS was increased to 100 μg/ml. One month after transfection, d2EGFP high-expressing cells (top 0.1–2% of the total population) were sorted using a FACSAria instrument (Becton Dickinson) and seeded directly in 96-well culture plates at a density of 1–10 cells per well. The cells were further cultured for 2 to 3 weeks, and then examined by epifluorescence microscopy. Wells containing a colony consisting of homogeneous cells emitting bright green fluorescence were identified, and each of these colonies was further grown and used to construct the secondary library.

### Construction of *E*. *coli* secondary library and screening

Genomic DNA was isolated from CHO transfectants that expressed high levels of d2EGFP fluorescence (see previous section). The human genomic insert was PCR-amplified using the In-Fusion AscI-S primer and Blend Taq-Plus- DNA polymerase (Toyobo). The product was inserted into *Asc*I-linearized pΔBM-d2EGFP-AscI using the In-Fusion HD Cloning Kit, and then transfected into competent *E*. *coli DH5*α cells. Examination of the insert DNA by PCR, isolation of plasmid DNA, and re-transfection into CHO DG44 cells were performed as described for the primary library. d2EGFP fluorescence from stable transformants was analyzed by flow cytometry using a FACSCalibur instrument (Becton Dickinson and Company) about 1 month after transfection, and tentatively positive secondary libraries were identified.

### Cloning and sequencing of genomic fragments

Plasmid DNA of the tentatively positive secondary libraries were transfected into *E*. *coli* strain SURE 2 (Agilent Technologies), and the resultant colonies were subjected to PCR amplification using Tks Gflex DNA polymerase (TaKaRa) to identify and group the inserts. To determine nucleotide sequences, inserts were amplified using the BigDye Terminator v3.1 Cycle Sequencing Kit (Applied Biosystems) using a set of primers 19 or 21 nt long derived from the vector pΔBM-d2EGFP-AscI. The products were analyzed on an ABI PRISM 310 genetic analyzer (Applied Biosystems). The sequences obtained were localized on the human genome using *Homo sapiens* (human) Nucleotide BLAST (NCBI; http://blast.ncbi.nlm.nih.gov/Blast.cgi?PAGE_TYPE=BlastSearch&BLAST_SPEC=OGP__9606__9558).

### Re-cloning of the B-3-31 sub-fragment

Six sub-regions of the B-3-31 sequence (3–1 to 3–3 and B1 to B3) were PCR-amplified. Each of the left and right 35 nt primers had a 20 nt target sequence at its 3’ end and a 15 nt vector sequence at its 5’ end. The bent and unbent sequences were combinations of three subparts of B-3-31. To clone these sequences, the middle subpart was amplified using a set of 20 nt specific primers, whereas the left and right parts were amplified using 35 nt primers. The latter primers had a 20 nt target sequence at their 3’ ends and 15 nt sequence at their 5’ ends derived from either the vector or middle subpart. The PCR products were recombined and inserted by InFusion reaction into the *Mlu*I site of pΔBM-d2EGFP plasmid. The resultant plasmids were transfected into *E*. *coli* DH5a, purified, transfected into CHO DG44 cells, selected with BS, and analyzed by flow cytometry as described above.

### Sequence analysis

Repetitive sequences in the human genome were analyzed using the “RepeatMasker” Web Server, made available by the Institute for System Biology (http://www.repeatmasker.org/cgi-bin/WEBRepeatMasker). The denaturation energy plot and stress-induced duplex destabilization (SIDD) [20] were calculated using the SIDD/ZDNA Analysis tool web server, made available by Dr. Craig. J. Bentham at the UC Davis Genome Center (http://benham.genomecenter.ucdavis.edu/sibz/). MAR potential was predicted using MAR-Wiz (http://genomecluster.secs.oakland.edu/marwiz/). Non-B DNA structure was predicted using non-B DB (https://nonb-abcc.ncifcrf.gov/apps/site/default). Furthermore, nucleosome occupancy [21], inverted repeats (repeat unit, more than 6 bp; spacer, 0–6 bp; entire length, more than 15 bp [22–24]), and end-to-end distance in three dimensions (3D) [25] were determined using the software described in each reference.

### FISH

Metaphase chromosome spreads were prepared from the indicated cells, and the locations of plasmid sequences were determined by FISH using a digoxigenin (DIG)-labeled probe prepared from the plasmid. Probe preparation and FISH procedures were described previously [9]. The slide was counterstained with propidium iodide and examined using an epifluorescence microscope (ECLIPSE TE2000-U, Nikon) equipped with a 100× objective lens (Nikon Plan Fluor, NA 1.30 oil) and an appropriate filter set. Digital images were acquired with a Nikon D7000 digital camera and processed with Adobe Photoshop CS6 (Adobe Systems, Inc.).

## Results

### Cloning of a human genomic sequence (B-3-31) that increases gene expression from amplified sequence

**Fig 2:**
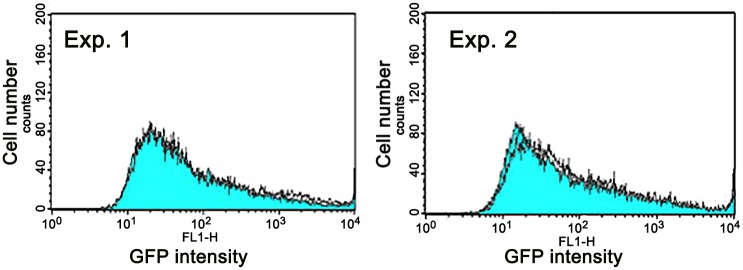
Effect of a palindrome sequence on gene expression from the amplified structure. Effect of a 2 × 25 bp palindrome on gene expression from the repeated sequence. pΔBM-d2EGFP-AscI (containing the palindrome) or pΔBM-d2EGFP (no palindrome) was transfected into CHO DG44 cells. Transformants were selected with blasticidin for about 1 month, and then analyzed by flow cytometry. Results from two independent transfections are shown. Each panel shows the pΔBM-d2EGFP cell population (blue filled line) that overlapped with the pΔBM-d2EGFP-AscI cell population (unfilled line).

Fig 1 schematically depicts library construction and screening. We constructed the human genomic library using *E*. *coli* host cells and the vector pΔBM-d2EGFP-AscI, which contains an IR sequence from the *DHFR* locus; the IR contains an internal sequence with MAR activity [4]. The plasmid pΔBM-d2EGFP-Asc I contains a 2 × 25 bp palindromic sequence at a restriction site (*Mlu*I) located immediately upstream of the promoter that drives *d2EGFP*. This sequence was used for insertion of random genomic fragments via the In-Fusion reaction, but the palindrome disappeared after insertion. Because palindromes are implicated in various biological processes, including transcription [26,27], we examined the effect of this palindrome by comparing pΔBM d2EGFP-AscI (with the palindrome) with pΔBM d2EGFP (without the palindrome). Fig 2 shows the results from two independent experiments. Stable transfectants exhibited almost identical levels of green fluorescence regardless of the presence or absence of the palindrome. In other words, the palindrome had very little effect on gene expression from an amplified tandem array generated by the IR/MAR method.

The *E*. *coli* primary library consisted of seven sub-libraries (A to G) containing about 220,000 independent colonies, 45% of which contained inserts from the human genome (average length, 1.27 kb). Thus, a simple calculation suggests that the library covered about 100 Mb of the human genome. Because sequences that control large genome domains, such as IRs, MARs, insulators, and locus control regions, appear roughly once per 100 kb, we assumed that this coverage would be sufficient to identify the sequences of interest. Plasmid DNA from the library was transfected into CHO DG44 cells, in which IR/MAR plasmids multimerize at extrachromosomal sites and then integrate into chromosome arms as short HSRs [8,10]. Such short HSRs can be further elongated to form fine ladder—type HSRs [8,14]. In this study, FISH confirmed that the plasmid DNA was amplified as expected (see next section). After selection of the transfectants with BS for about 1 month, we isolated cells emitting high levels of green fluorescence (top 0.1–2% of the total population) by FACS and examined them by fluorescence microscopy.

From 25 clonal or sub-clonal CHO cells that expressed high levels of d2EGFP, we recovered the human genomic insert by PCR and constructed 25 secondary *E*. *coli* libraries (i.e., one for each of the 25 clones). This step was necessary because any DNA co-transfected with an IR/MAR plasmid is co-amplified in the cell [3,11,14]; thus, plasmids with different inserts could be co-amplified in the same clone. These secondary libraries contained inserts with an average length of 1.71 kb, longer than in the primary library.

We transfected DNA from each secondary library, along with vector pΔBM-d2EGFP-AscI as a control, into CHO DG44 cells (Exp. 1–3 in S1 Fig). Subsequently, we chose six sub-libraries in which the transformants emitted brighter fluorescence than the control, and classified these as tentatively positive (highlighted in red in S1 Fig). Plasmid DNA from these secondary libraries was re-transfected into CHO DG44 cells (S1 Fig, Exp. 4), and four of the six candidates were reproducibly positive.

**Fig 3:**
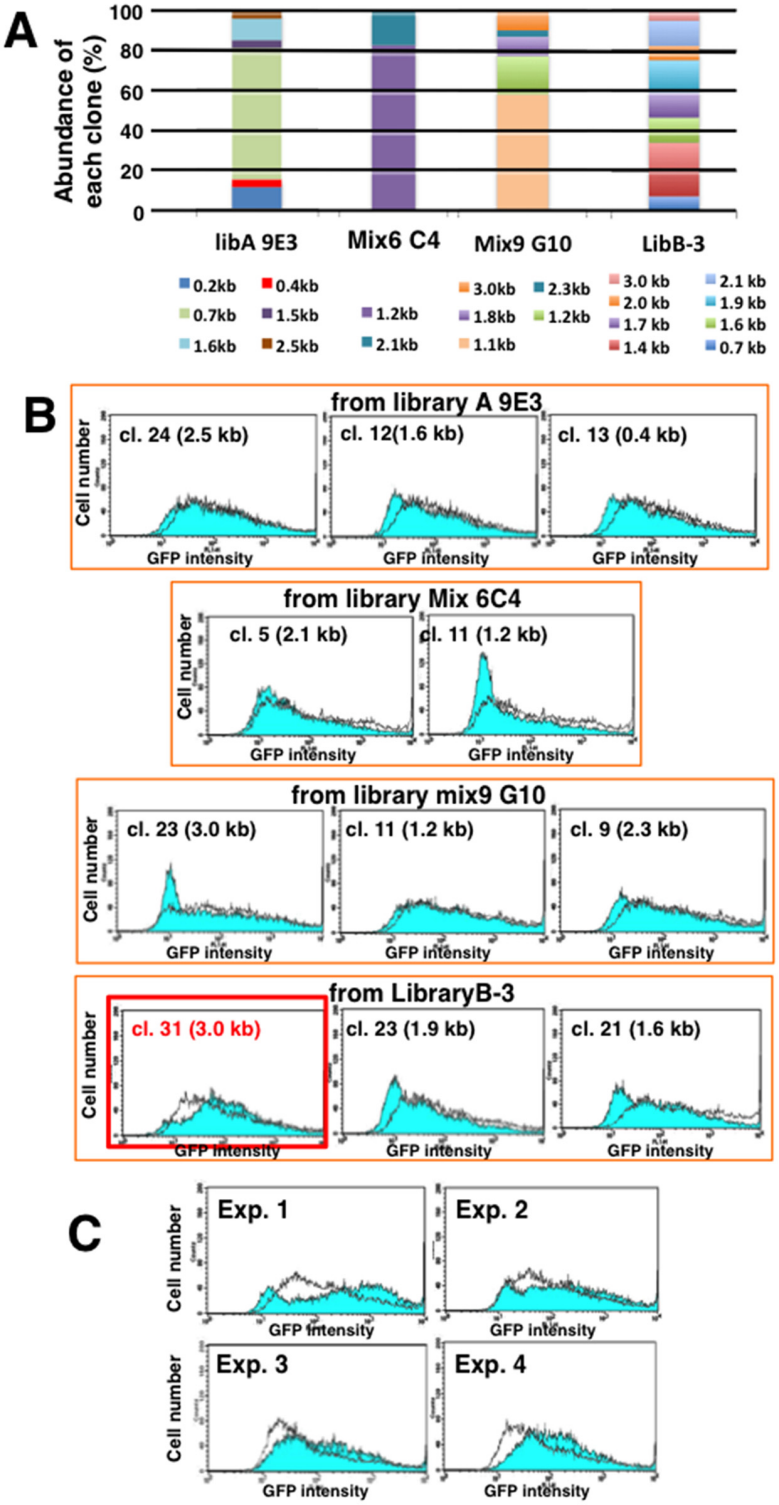
Cloning of the B-3-31 sequence. Plasmids from the positive secondary library were transfected into *E*. *coli*. Colony PCR followed by restriction enzyme digestion/gel electrophoresis revealed the composition of each secondary library (A). DNA from each clone was transfected into CHO DG44; transformants were selected for 1 month, and then analyzed by flow cytometry. In each panel, the test cell population (vector plus insert: blue filled line) overlaps with the control cell population (vector alone: unfilled line). Representative results from 11 of 21 clones examined are shown in B, and the remaining results appear in S1 Fig. Clone 31 from library B-3 (B-3-31) increased d2EGFP expression to the greatest extent, and this effect was reproduced in four independent transfections by three investigators (C).

Plasmid DNA from these secondary libraries was cloned into *E*. *coli* cells. The composition of each library was determined according to insert length (Fig 3A), revealing that each secondary library had two to eight distinct human genomic inserts. We then transfected the plasmid DNA from each of 21 clones into CHO DG44 cells, selected transformants, and analyzed d2EGFP expression as before (Fig 3B and S2 Fig). Ultimately, we chose clone 31 from library B-3 (B-3-31) because it expressed the highest level of d2EGFP.

The elevation of d2EGFP expression by B-3-31 was highly reproducible: this sequence consistently increased expression relative to the control IR/MAR vector (pΔBM-d2EGFP) in more than eight independent rounds of transfection and evaluation. Four representative results are shown in Fig 3C, and other results appear in a later section, in which we describe the characterization and dissection of B-3-31.

### The B-3-31 sequence increases gene expression without influencing gene amplification

**Fig 4:**
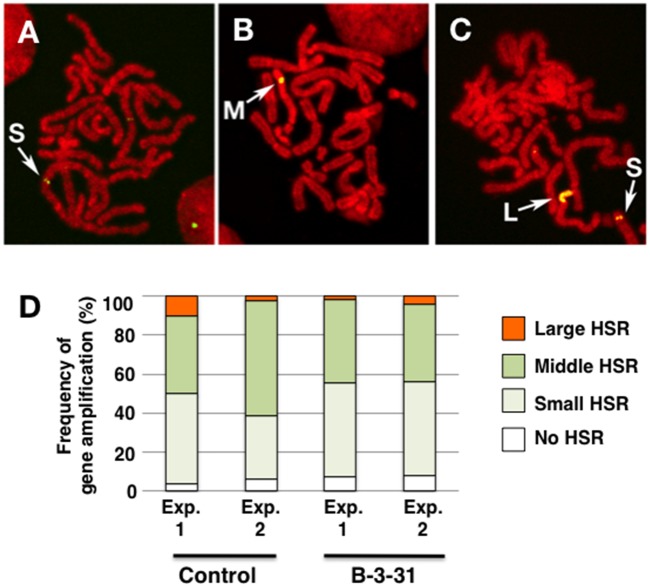
Effect of B-3-31 on IR/MAR-mediated gene amplification. Metaphase chromosome spreads prepared from stable transformants were analyzed by FISH using a plasmid-derived probe. Representative images of small (S; panel A and C), medium (M; panel B), and large (L; panel C) HSRs are shown. The frequencies of cells bearing these HSRs among transformants from the control pΔBM-d2EGFP-AscI plasmid and the same plasmid containing the B-3-31 sequence were determined by examining more than 30 metaphase cells in each case from two independent transfections (panel D). If more than two HSRs were present in a cell, we counted it as a single cell bearing a HSR of the largest size.

We next asked whether B-3-31 increased d2EGFP expression by increasing gene amplification or promoting transcription from the amplified gene. To this end, we prepared metaphase chromosome spreads from stable CHO DG44 transformants 1 month after transfection and detected the plasmid sequence by FISH (Fig 4). The extent and efficiency of gene amplification were similar between the control pΔBM-d2EGFP-AscI plasmid and the same plasmid containing the B-3-31 sequence (Fig 4D), and these results were reproducible when we characterized and dissected B-3-31 (see below). Therefore, we concluded that B-3-31 increased gene expression from the amplified sequence without influencing the amplification process.

### The B-3-31 sequence alleviates the epigenetic silencing that occurs at the amplified array on the IR/MAR plasmid

**Fig 5:**
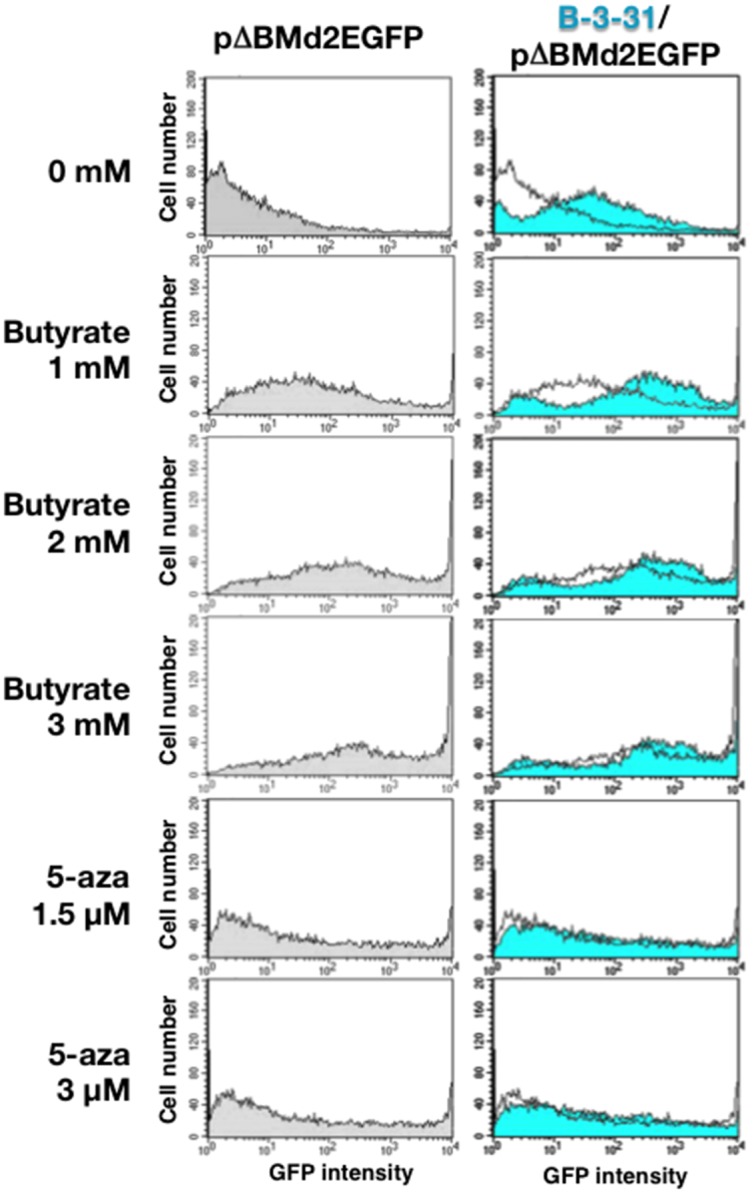
Effect of butyrate or 5-azacytidine on expression from the amplified plasmid array. Thirty days after transfection with pΔBM-d2EGFP with or without B-3-31 into CHO DG44 cells, the cells were treated with the indicated concentration of butyrate or 5-azacytidine. After 3 days, d2EGFP expression was analyzed by flow cytometry. Charts on the left show the results obtained with pΔBM-d2EGFP cells, and charts on the right show the results obtained with cells harboring pΔBM-d2EGFP with B-3-31 (blue filled line) that overlapped with those harboring pΔBM-d2EGFP (unfilled line).

Thirty days after transfection with the IR/MAR vector (pΔBM-d2EGFP) with or without the B-3-31, we treated the cells with various concentrations of butyrate or 5-azacytidine, which are known to increase histone deacetylation and decrease DNA methylation, respectively. After 3 days, we analyzed d2EGFP expression by flow cytometry. The results (Fig 5, left charts) showed that these drugs considerably increased d2EGFP expression in the cells transfected with pΔBM-d2EGFP. The increase in expression was greater with butyrate than with 5-azacytidine. The results confirm our previous conjecture that expression from the IR/MAR plasmid amplified in human COLO 320 cells might be increased by treatment with butyrate and/or 5-azacytidine [28]. Consistent with the above result, the inclusion of the B-3-31 sequence in the plasmid resulted in increased expression even when the cells were cultured in the absence of drug (Fig 5, uppermost charts). Therefore, B-3-31 may increase the level of gene expression silenced by an epigenetic mechanism involving histone acetylation and DNA methylation. Notably, a low concentration of butyrate (1 or 2 mM) increased d2EGFP expression in cells harboring the vector with B-3-31 to a greater extent than in cells harboring the vector alone. We suggest that the plasmid with B-3-31 underwent amplification and adopted a structure whose silencing could be more easily alleviated.

### Characterization and dissection of the B-3-31 sequence

**Fig 6:**
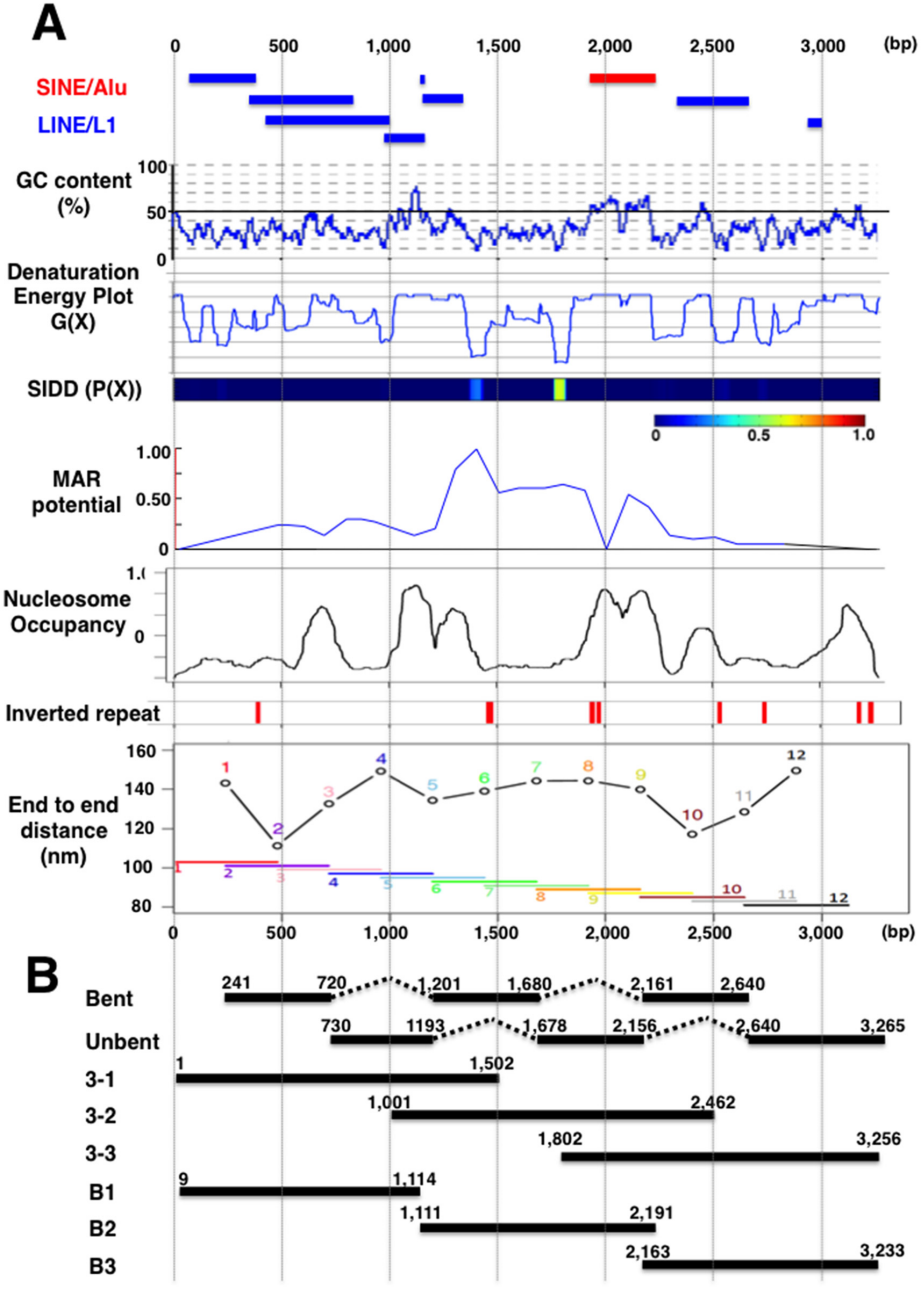
Characteristics of B-3-31 sequence, and the dissection strategy. (A) The panels show the locations of highly repetitive sequence (LINE and SINE), GC content, denaturation energy, locations of stress-induced duplex destabilization (SIDD) regions, predicted MAR potential, predicted nucleosome occupancy, inverted repeats, and 3D end-to-end distance of 480 bp sliding windows along the B-3-31 sequence. (B) To determine which region is responsible for elevation of gene expression, the indicated sub-regions were re-cloned and examined.

Sequencing revealed that B-3-31 is a 3,271 bp sequence from *Homo sapiens* chromosome 2p16.1 (57,245,485 to 57,248,755 of Gen Bank accession NC_018913.2; S3 Fig). The region is gene-poor, containing only one pseudogene (*EIF2S2P7*) within 1 Mb of B-3-31. Therefore, B-3-31 is unlikely to act as a promoter or enhancer. Furthermore, B-3-31 contained no significant sequence motifs, including the consensus binding sequence for insulator-binding CTCF zinc-finger proteins. On the other hand, B-3-31 contained sequences derived from Alu-type SINEs and L1-type LINEs (Fig 6A), which together constituted 1,972 bp (60.29%) of the sequence. The A/T content was very high throughout B-3-31 (Fig 6A), with an average value of 67.8%. The frequency of CpG dinucleotide was also low, and it appeared only 14 times throughout the 3,271 bp sequence.

Various physical properties of DNA influence gene expression. Therefore, we calculated the denaturation energy and predicted SIDD regions by SIDD/Z-DNA analysis (Fig 6). Because various types of non-B DNA structures also influence gene expression [24], we searched for inverted repeats that could form cruciform structures [22–24], predicted nucleosome occupancy [21], and calculated the 3D end-to-end distance of 480 bp sequences in sliding windows along B-3-31 to identify bent DNA structures (DIAMOD [25]). The results of these analyses are summarized in Fig 6A.

**Fig 7:**
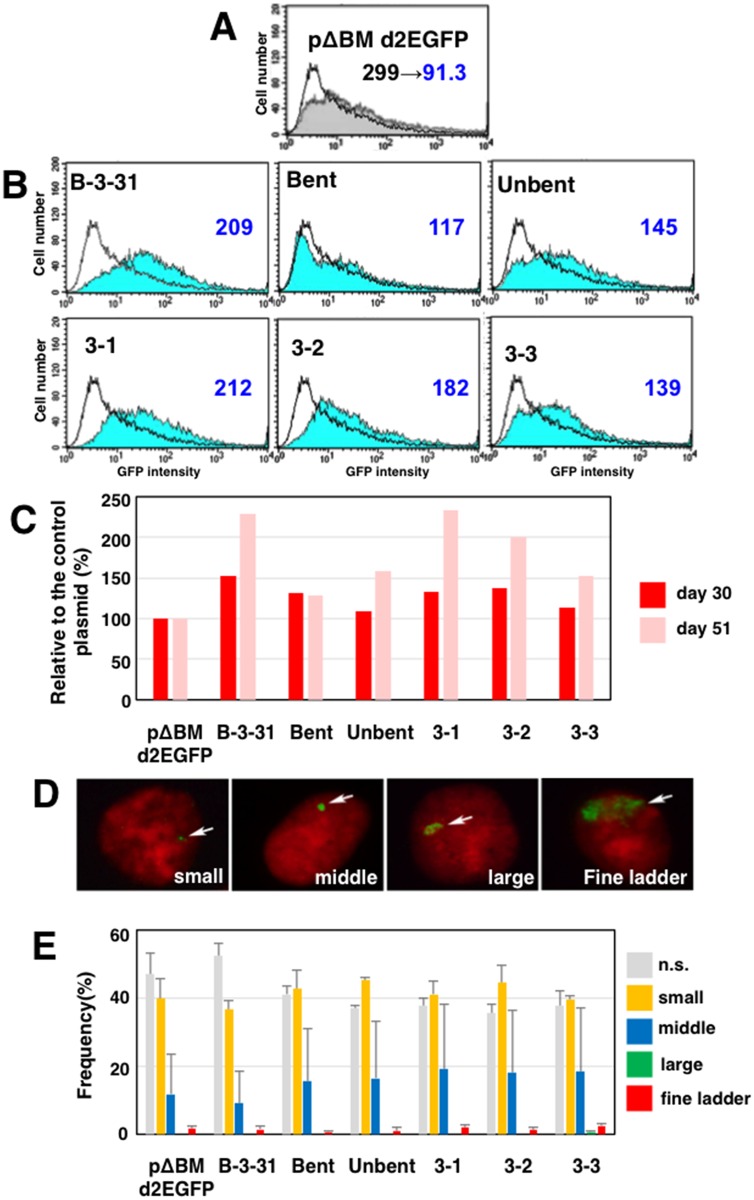
Dissection of B-3-31 (1). The sub-regions of B-3-31 (bent, unbent, 3–1, 3–2, and 3–3; Fig 6B) were re-cloned into the *Asc*I site of the IR/MAR vector (pΔBM-d2EGFP-AscI). The resultant plasmids and the control plasmid pΔBM-d2EGFP were transfected into CHO DG44 cells, and transformants were selected with BS. The results of flow-cytometric analysis of d2EGFP expression 30 and 51 days after transfection with the control pΔBM-d2EGFP are shown in A. In the chart, the cell population at 30 days (gray filled line) overlaps with the cell population at 51 days (unfilled line). Changes in average GFP intensity can be observed in the chart. At 51 days, the results of flow-cytometric analysis of d2EGFP expression in various cell populations are shown in B. In each chart, the test cell population (blue filled line) overlaps with the control cell population (pΔBM-d2EGFP; unfilled line). Average GFP intensities were observed for test cells, which can be compared with those of control cells at 51 days (panel A; 91.3). At days 30 and 51 after the transfection, average GFP intensities were divided by that of the control population, and the results are plotted in C. At day 29, cells were fixed and analyzed by FISH using a plasmid-derived probe. To analyze many cells, we evaluated gene amplification of the plasmid in interphase nuclei. Typical FISH images appear in D. Using these images as a standard, the frequency of each type of amplification was scored by examination of more than 300 nuclei in triplicate; the means +/- standard deviations are plotted in D.

To determine which sequence inside B-3-31 might increase gene expression from tandem repeats, we re-cloned ~1,500 bp fragments from various regions of B-3-31 into pΔBM-d2EGFP (Fig 7B; 3–1 3–2 and 3–3). In particular, we noted regions that were predicted to be bent (Fig 6B), and assembled and re-cloned sequences from three regions including two strongly bent regions (bent) and three unbent regions (unbent). The resultant plasmids were transfected into CHO DG44 cells, selected, and analyzed by flow cytometry as described above. Analyses at 30 and 51 days after transfection revealed a reduction in d2EGFP expression at these intervals in cells transfected with pΔBM-d2EGFP (Fig 7A), which is consistent with the conjecture that these amplified genes had been subjected to silencing. As before, B-3-31 increased expression compared with the vector pΔBM-d2EGFP (Fig 7B). However, both bent and unbent sequences slightly increased expression, and this was also the case for fragments 3–1, 3–2, and 3–3. These observations suggested that B-3-31 does not contain a “core” sequence responsible for the elevation of gene expression. Similar results were obtained in two additional independent transfections and subsequent analyses (data not shown).

Notably, gene expression from plasmids bearing these inserts relative to the parent vector was usually higher 51 days after transfection than at 30 days (Fig 7C), suggesting that B-3-31 or its fragments prevented the epigenetic gene silencing that occurs in repeated transgenes over the course of culture. FISH analysis of these transformants (Fig 7D and 7E) suggested that the introduced plasmid was amplified similarly to the vector pΔBM-d2EGFP.

**Fig 8:**
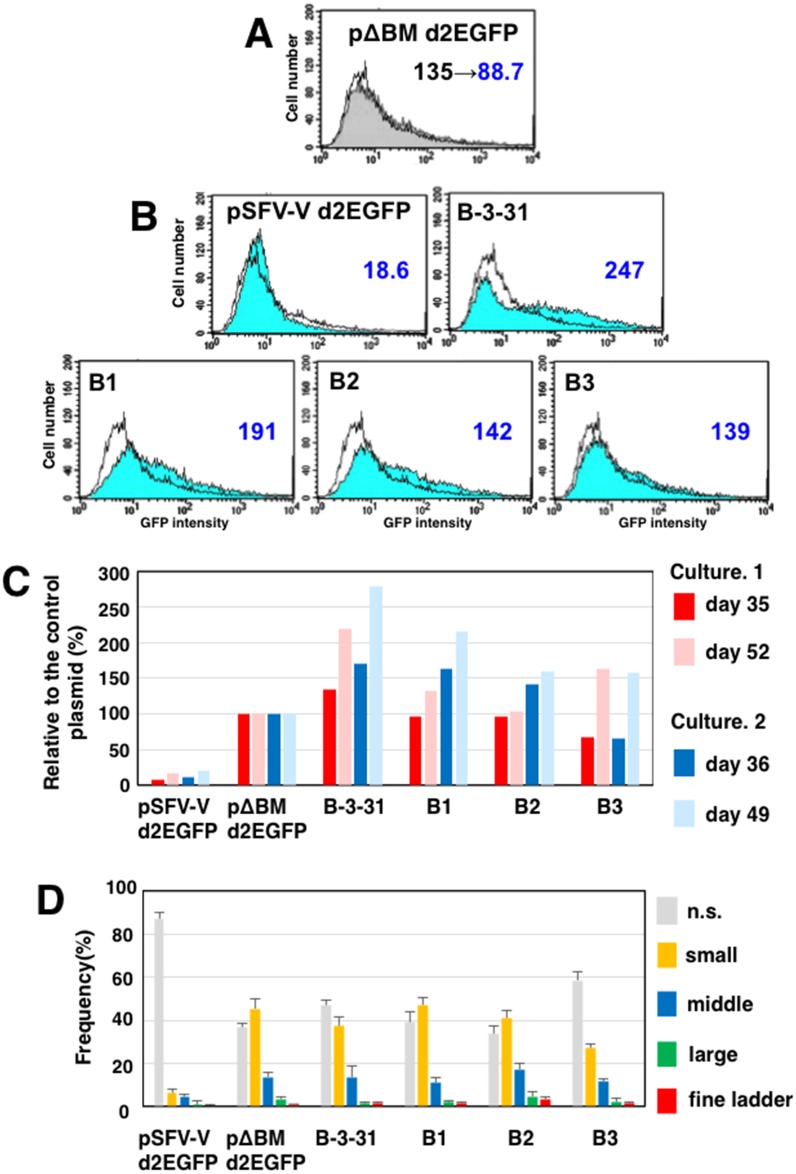
Dissection of B-3-31 (2). The B1, B2, and B3 sub-regions of B-3-31 (Fig 6B) were re-cloned into the *Asc*I site of the IR/MAR vector (pΔBM-d2EGFP-AscI). The resultant plasmids and control plasmids with (pΔBM-d2EGFP) or without (pSFV-V d2EGFP) the IR/MAR were transfected into CHO DG44 cells, and transformants were selected with BS. The results of flow-cytometric analysis of d2EGFP expression at 36 and 49 days after transfection with the control pΔBM-d2EGFP plasmid are shown in A. In the chart, the cell population at 36 days (gray filled line) overlaps with the cell population at 49 days (unfilled line). At 49 days, the results of flow-cytometric analysis of d2EGFP expression in various cell populations are shown in B. In each chart, the test cell population (blue filled line) overlaps with the control cell population (pΔBM-d2EGFP; unfilled line). Average GFP intensities are noted for test cells, which can be compared to control cells at 49 days (panel A; 88.7). For two independent cultures from the same transfection, average GFP intensities obtained at the indicated times were divided by that of the control population (C). At day 31, the cells were fixed and analyzed by FISH using a plasmid-derived probe. The frequency of each type of amplification (representative image appears in Fig 7D) was scored by examining more than 300 nuclei in triplicate; the means +/- standard deviations are plotted in D.

The region from nucleotides 1,250 to 2,100 of B-3-31 was predicted to have significantly higher MAR activity than the rest of the sequence (Fig 6A). This region contained two short regions with high SIDD scores and two prominent inverted repeats, and also had a lower probability of nucleosome occupancy. These features might be responsible for the anti-silencing effect. Furthermore, the region spanning nucleotides 1,300–1,900 was unique and lacked repetitive SINEs or LINEs. Every region examined in Fig 7 contained all or part of this sequence, potentially explaining why all of those regions enhanced expression. Therefore, in light of this observation, we hypothesized that a core region responsible for anti-silencing activity of the B-3-31 might indeed be present in this region. We explored this possibility by constructing plasmids containing fragments B1, B2, and B3 (Fig 6B) in vector pΔBM-d2EGFP as before. Because this vector contains an IR/MAR sequence from the *DHFR* locus, we used an additional control plasmid (pSFV-V-d2EGFP) that is identical to pΔBM-d2EGFP but lacks the IR/MAR sequence. These plasmids were transfected to CHO DG44 cells, selected, and analyzed as described above. pSFV-V-d2EGFP exhibited only a low level of gene expression. As expected, because pSFV-V-d2EGFP is a normal plasmid that does not contain an IR/MAR, it was not amplified in cells (Fig 8). By contrast, the plasmid with the IR/MAR sequence (pΔBM-d2EGFP) was amplified to various extents, and exhibited higher levels of d2EGFP expression (Fig 8). The expression of d2EGFP in these cells decreased from day 36 to day 49, suggesting again silencing of the amplified genes (Fig 8A). By contrast, insertion of B-3-31 into pΔBM-d2EGFP further increased expression 35–52 days after transfection, whereas gene expression from plasmids containing fragments B1, B2, or B3 expressed higher levels of d2EGFP than the parent vector but lower levels than the plasmid containing B-3-31. Importantly, the expression level of plasmids containing B1, B2, or B3 did not differ significantly, once more suggesting that B-3-31 contains no core sequence responsible for increasing expression from the amplified sequence.

In both of these independent experiments, the effect of B-3-31 (or its fragments) on gene expression always increased as the cells were cultured for longer periods of time (Fig 8C), providing further evidence that the effect of B-3-31 on gene expression was due to alleviation of epigenetic silencing.

## Discussion

When genes are amplified during malignant transformation, the number of genes per cell is generally roughly proportional to the amount of protein produced [28–30]. By contrast, genes amplified by artificial methods such as the IR/MAR approach [28] are often silenced because the amplicons are simple repeats. Silencing of simple repeats is necessary for genome maintenance. For example, silencing of at least a portion of a highly repeated array of ribosomal DNA is required for the genome stability [31]. Consistent with this, forced expression of amplified genes on a HSR results in fragmentation of the HSR [28]. IR/MAR plasmids generate both tandem and inverted repeat sequences [7] in cells. Transcription of an inverted repeat generates double-stranded RNA that silences the original DNA sequence via the RNAi pathway. On the other hand, direct repeats are silenced through the RIGS pathway, as described above. As noted, genes amplified by the IR/MAR method have histone modifications specific to heterochromatin [8,16]. In this study, using a novel screening system involving IR/MAR gene amplification, we isolated a genomic sequence (B-3-31) that at least partially alleviates such silencing. Below, we discuss how B-3-31 might exert its anti-silencing activity.

Various non-B DNA structures, especially those near transgene promoters, regulate gene function [32]. One example is the cruciform structure generated from a palindromic sequence, which can influence multiple gene functions including transcription [26,27]. Palindromic sequences also accelerate the gene amplification process [7,33,34]. In this study, however, a 2 × 25 bp palindrome upstream of the promoter did not exert anti-silencing activity (Fig 2). Bent DNA structures are also implicated in transcriptional activation [35,36]. Although B-3-31 had two regions in which the DNA strand was predicted to bend significantly, fine-scale studies of the sequence ruled out the possibility that these structures were responsible for anti-silencing.

Several types of sequence elements elevate gene expression, and recent studies compared them [37,38]. These elements include an insulator element that blocks the effect of surrounding chromatin, thereby acting as a barrier sequence against heterochromatin spreading. The insulator is bound by a CTCF zinc-finger protein, which, in collaborating with cohesin protein, generates a chromatin loop that is independent from other genomic regions [39]. The most studied example of this phenomenon is the chicken beta-globin 5’-HS4 insulator, which binds CTCF, exhibits MAR activity, and has a core sequence of only 247 nt [40]. However, the anti-silencing activity of B-3-31 was not restricted to such a short region (Figs 7 and 8), and B-3-31 does not contain a consensus sequence for CTCF binding (CCGCGNGGNGGCAG). Therefore, the insulator cannot plausibly explain the activity of B-3-31.

Multiple studies show that MARs increase transgene expression, and consequently these sequences have been used to boost recombinant protein production [41–43]. The IR/MAR plasmid vector we used contains a sequence within the *DHFR* IR that exhibits *in vitro* MAR activity [4]. Like B-3-31, MARs are usually A/T-rich, but they tend to be much shorter than the anti-silencing sequence we identified. B-3-31 does contain a region with potential MAR activity, but its anti-silencing activity was not restricted to that region. Therefore, B-3-31 activity does not depend on a MAR.

STabilizing Anti-Repressor (STAR) elements were identified in a screen for human genomic fragments that prevent heterochromatin spreading [44]. However, these sequences are not A/T-rich and are conserved between human and mouse. Because B-3-31 was highly A/T-rich throughout its entire sequence and is not conserved in the mouse genome, B-3-31 is not a STAR element.

Ubiquitous chromatin opening elements (UCOEs) are regulatory elements derived from promoter-containing CpG islands of ubiquitously expressed housekeeping genes [45]. The core CpG island element (IE) from hamster adenine phosphoribosyltransferase effectively prevents DNA methylation [46]. However, B-3-31 is clearly unrelated to these elements, both because it is derived from a gene desert and because it is CpG-poor.

**Fig 9:**
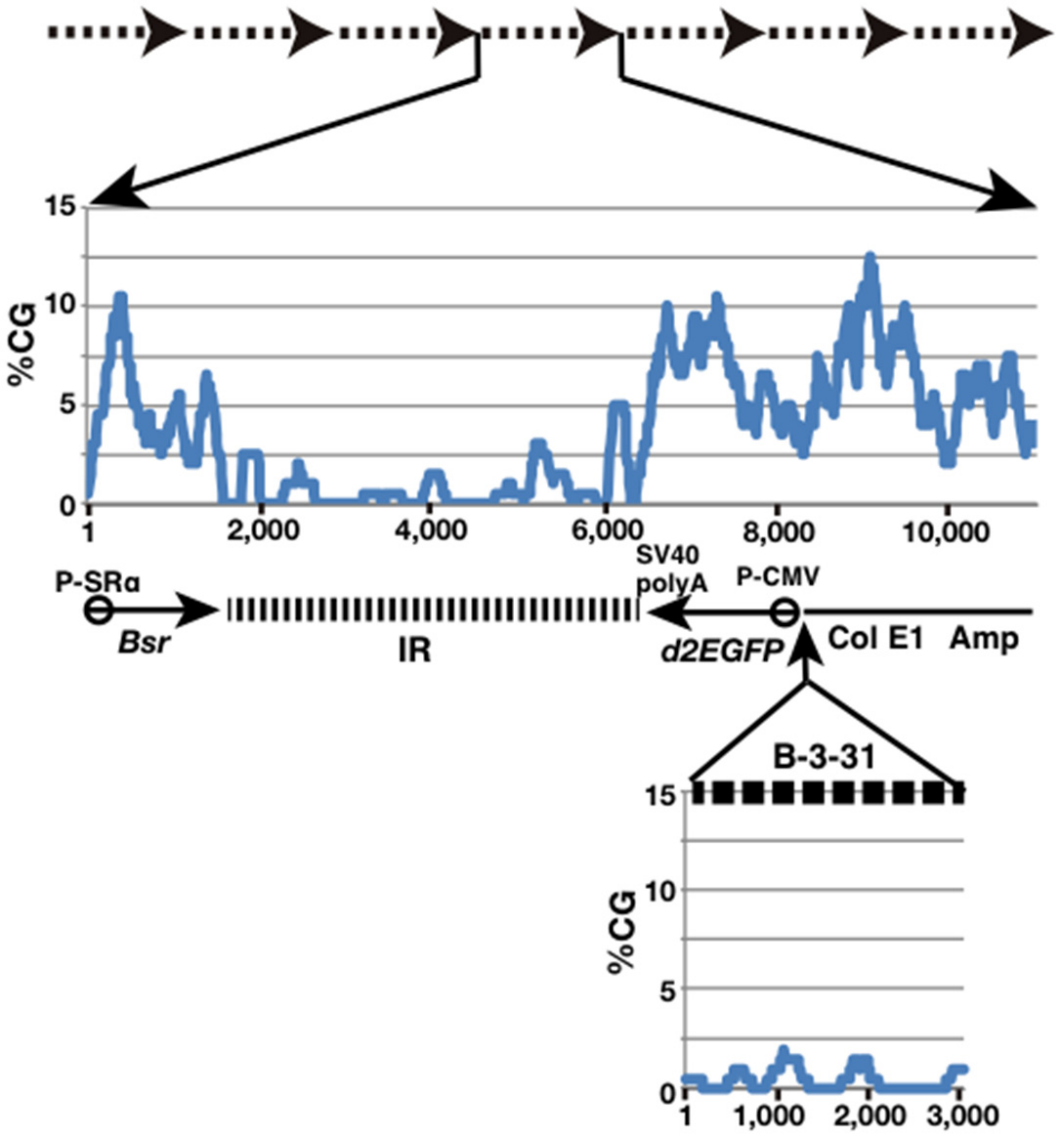
Frequency of the CpG dinucleotide along the amplified plasmid array. The pΔBM-d2EGFP plasmid is amplified as a direct repeat, because it is an IR/MAR plasmid (dashed arrows above). The frequency of the CpG dinucleotide along the pΔBM-d2EGFP plasmid is plotted at the middle panel. The frequency of CpG along the B-3-31 sequence is shown in the lower panel, and it was inserted into pΔBM-d2EGFP plasmid at the position indicated in the figure.

In light of our observations, the anti-silencing activity of B-3-31 cannot be attributed to a sequence that forms a non-B structure or previously reported sequence elements that alleviate epigenetic chromatin silencing. Instead, our data suggest that its activity is scattered throughout its entire 3,271 bp sequence. The only features of B-3-31 that persist over its whole length are its A/T-richness and low abundance of CpG. CpG is a target of DNA methylation, a stable landmark for heterochromatin formation through histone modification and recruitment of heterochromatin proteins [47]. Therefore, we hypothesized that insertion of a CpG-poor sequence greater than 3 kb in length might prevent heterochromatinization. Fig 9 (middle panel) shows the frequency of the CpG dinucleotide along the vector pΔBM-d2EGFP. The sequence was amplified as a direct repeat, as is usually the case for IR/MAR plasmid amplification (upper panel). The IR is also CpG-poor, but is located downstream of the d2EGFP gene, where it is likely to be ineffective. By contrast, in the original vector configuration, the CpG content is quite high upstream of d2EGFP, and insertion of a long CpG-poor sequence such as B-3-31 (lower panel) might be effective in anti-silencing activity. The A/T-richness might also facilitate denaturation of the DNA duplex, and thus might act in concert with the low abundance of CpG.

In summary, we isolated a novel genomic region that alleviates the silencing of a repeated amplified sequence. This region belongs to a novel category of sequence that prevents epigenetic chromatin silencing, and its activity may be a simple consequence of its A/T-richness and low abundance of CpG. In this study, we focused only on expression from the amplified sequence. However, B-3-31 may also be effective on conventional vectors that do not undergo amplification, because transgenes in general are a frequent target of silencing. The B-3-31 sequence could be used in combination with a gene amplification strategy such as the IR/MAR method to improve recombinant protein production.

## Supporting Information

S1 Fig Screening of the libraries. Plasmids from 25 independent secondary libraries were transfected into CHO DG44 cells in three separate experiments (Exp. 1 to 3). Six positive secondary libraries (marked as “+” and boxed in red) were re-transfected into CHO DG44 cells (Exp. 4). In each experiment, pΔBM-d2EGFP-AscI was transfected in parallel as a control. After selection for 1 month, the cells were analyzed by flow cytometry. Results from control (unfilled line) and test (blue filled line) were overlaid.
(TIF)

S2 Fig Screening of cloned plasmids that constitute the positive secondary libraries. Experiments were performed as described in the legend of Fig 3; results not shown in Fig 3 appear here.
(TIF)

S3 Fig Sequence of B-3-31. (DOC)
