# Supplementary figures and images for: Cloning and Characterization of a Human Genomic Sequence that Alleviates Repeat-Induced Gene Silencing
Source: PLoS One. 2016 Apr 14;11(4):e0153338. doi: 10.1371/journal.pone.0153338 (PMC4831671; doi:10.1371/journal.pone.0153338)

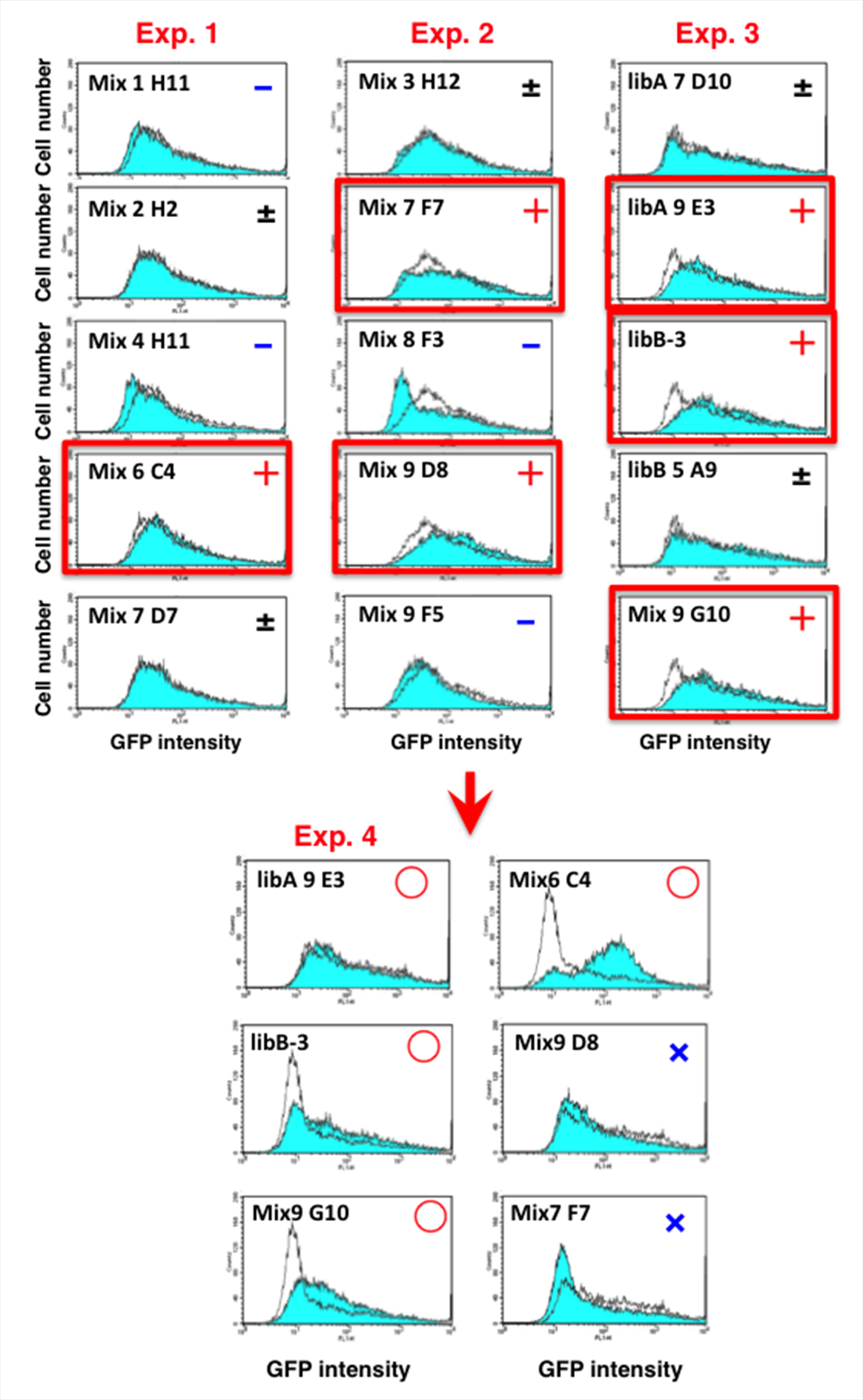

Supplement: S1 Fig — Plasmids from 25 independent secondary libraries were transfected into CHO DG44 cells in three separate experiments (Exp. 1 to 3). Six positive secondary libraries (marked as “+” and boxed in red) were re-transfected into CHO DG44 cells (Exp. 4). In each experiment, pΔBM-d2EGFP-AscI was transfected in parallel as a control. After selection for 1 month, the cells were analyzed by flow cytometry. Results from control (unfilled line) and test (blue filled line) were overlaid. (TIF) [file pone.0153338.s001.tif]

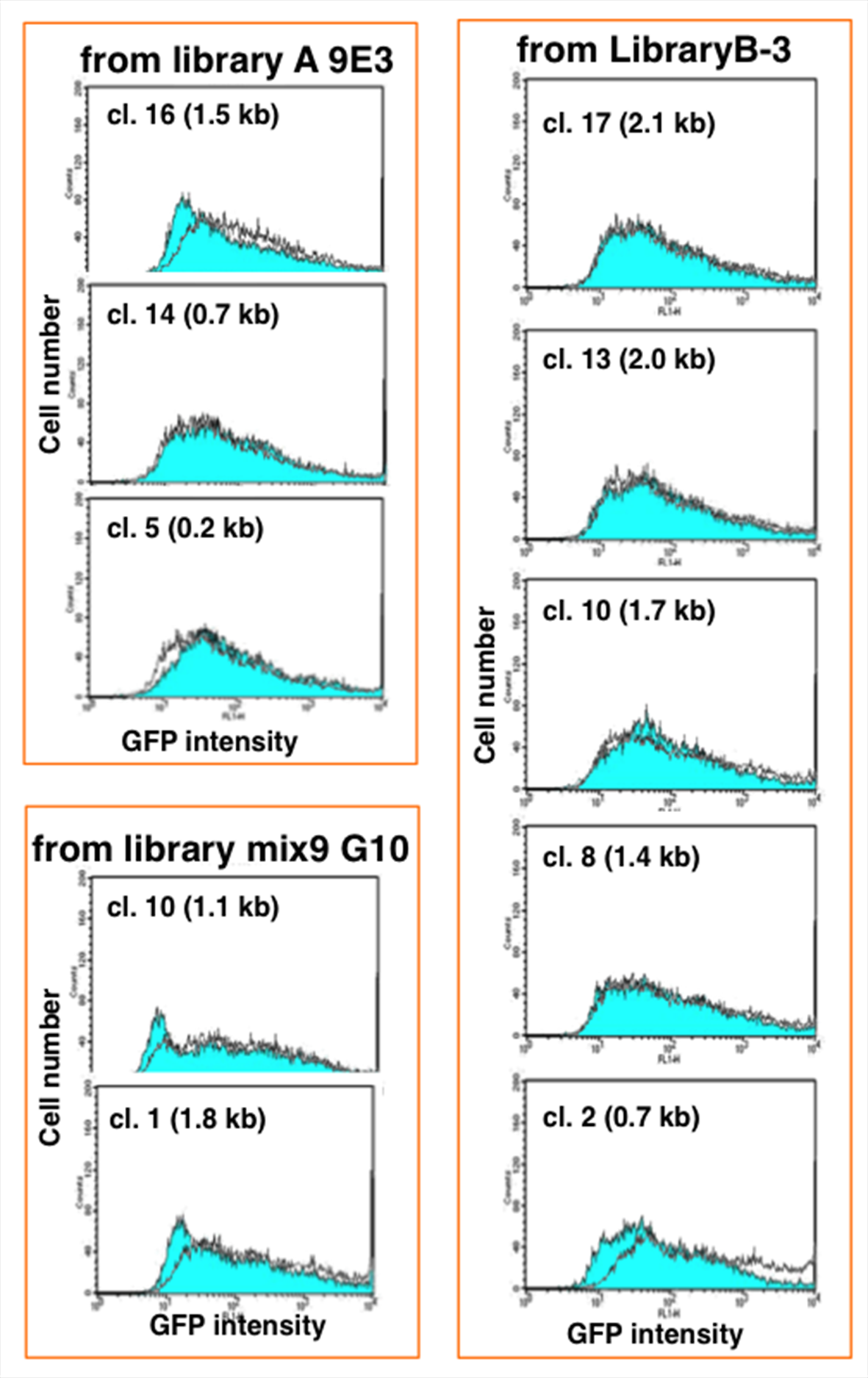

Supplement: S2 Fig — Experiments were performed as described in the legend of Fig 3; results not shown in Fig 3 appear here. (TIF) [file pone.0153338.s002.tif]
