# Supplementary material for: Cloning and Characterization of a Human Genomic Sequence that Alleviates Repeat-Induced Gene Silencing
Source: PLoS One. 2016 Apr 14;11(4):e0153338. doi: 10.1371/journal.pone.0153338 (PMC4831671; doi:10.1371/journal.pone.0153338)
Supplement: S3 Fig — (DOC) [file pone.0153338.s003.doc]

AAATTACCTACACTTCTGCAGCTAATTCTTCCCTCTGAACCAGCTTGCAGAACATTACATATGTGATTTTTGTCACTAGCTTAATTTGAATTTCTTAGAAATATACATTGAAATCAAATATTATATTCTCCTTTGTGTCCAGCTTTTTTCAGTTATTATAGTGTTTTTGAGATCTATTGATGTCGTTGCATGTATTTGTTGTTGGCTTATAATTTTTATTGATAATTAGTATTCAAATGTATTATCTATTACATCCTATTGATGTGCATTTTACTTGTTCTTGATTTTGGATATGACAAATAAAGTTACTATGAACATTATGTACATGTCTTCTTGTACATATATTTTTAAAAATGTATACTTAAATGTTGCCTGGTTTGGAAGCCATTTAATGAAATGGAATTTTATTTTGTGATTTTTATTTGGTAGTATATGGTGATGCAATTTGTGAAAAGTAATTTTAAGTACTGCTGTCTTGTGCAACACACTCAATTTTTAGTACCTTTTTTGTGGCTTCCTTAAGATTTTCCATTTATAAAAACTTGTTACTGCAAAAAAGAAAAATAAAATTCACTTCTTTTGACAAGCTGTATAATATGTAATTATTTGTCAAATTCATTTTTGCTAAGTACACTGGGTAAGACCCCTTTACAATGTCGAATAGCAGCAGCAAGTGTTGACATCCTTAACTTGTTATAAATTTTAGGTGGAAAGATACAGTATTTTGCCATTATGGCGCTAGTTTGTGGTTTTATCAAATGCCCTTTATTAGCTTGATGAATTTTCTTCTATGCTTAGTTTGTATAAATGAATGTTGAATTTTGTCAAATGCTTTTCTGCATCAAATGTAATCATTATATGTTTATTCTTTTGATTCTGTTTATATGGTGAAATGTTGAATAAATCTTGCTTAAATAAACTTTACTTGGTGAATAGATAGCATGTTTTTATCTTGTGGGACTTAATTTACTAGCATTTCTTTTTTTTTTAATTATACTTTAAGTTTTAGGTTACATGTGCCCAACATGCAGGTTAGTTTCATATGTATACATGTGTCATGTTGGTGTGCTGCACCCAGTAACTCGTCATTTAACATTAGGTATATCTCCAAATGCTGTCCCTCCCCACTCCCCCCACCCCACAACAGGCCCCGGTGATGGAATTGAACAATGAGAACACATGGACACAGGAAGGGGAACTTACTAGTATTTCACTAGGAATATTTACTTGTGTGCTTTTAAACGATATTGGTCTATAGTGTTTCTCTTATAACATCGTGTCTTGTTTGGTGTCAGACAGCAGCTAATGGCCCTATTAAATGAGTTGGGAAGTGTTTCTTGCTTCCTTAATACACGTAGACATTTAGTCAACAATGAATGCTAAATATTATTAGTTCTTTTGGAATATTTCAATGATTTTTAATATAAATTATATGACAAATTTTGGTCTCAGTGTATTATTTCCTTAATACACTACTGTATTTGCTAACATTTCATAATAATTGTTTTAATCTTTGTAATCCTAAGTAAGATTTGAAATCAGTGTCCTTTTGTGACATAAATTCTTCTTGGTTTCTATATTAACTAGAATTGTGCTTATAAGTTAAAAGTGTTTTTCTTCTTACATAATTAAGGGTTTTACAGCAGAAATTTTATTATCTGAATATAATTTCCTCTGTTACATAGATTTAGTTTTGGCATGTCCTTCTTTATTTTCTAGTTTGTATATTCTCTTTTGATTTCTTCTTTGATGCAATTGTGTAGTGTCCATTTAAATCATTAATTATATTGATTAATTTTTATTAGCAATTTCTAAATATACTTTTCTTGACCACACAGGTTGCTTGTAATAATGCACATATTTGGACTTGGTAAATGTGTTGTTTTCAATACATGGTCACATTTTATTTCCGTTTCTGTTTTTGTTCTTTTGAGACACAGTCTCACTGTGTCACCCAGTCTGGAGTTCAGTGGCATGAACTTGGCTCACTGCAAACTCCACCTCCTGGGTTCAAGCGATTCCCTTGCCTCAGCCTCCCGAGTAGCTGGGATTACAGGCCCACACCACCATGCCCAGCTAATTTTTGTATTTTTAATAGAGGCTGGGTTTTGCCATGTTGGGCAAGCTGGTCTCGAACTCCTAGCCTAAAGTGATCCTCCTGCCTTGGCCTACCAAAGTGCTGGGATCACAGGCATGAGCCACTGCACCTGGGCCATGGTCACATTTTGTATAAGTTTTACATATGTTTAACCAGTAATATATATTCTCTCTATAAATAATAAAAAGATGTATCCATGTGTGTTAATTTAAGATTATTGATTATATTACTCAATTTGTTTACATTTCAACTTCTTTTTCCTTGATCAGGTCTATCAAAATTTGAGCAAGAAATGTAGAAGTCTTAGAATTTTCACCAAAATGTGTTCAGGGATGTACATTTTTCATCACTCCAGCCTGGGAACCTGGTAAACCCCTTCAATATGGAGGCTCCCATTTTTCCTCAAGTAAGACAAATTTTCTTCTATATTTTGTTTAAATATTAGCTCTTCTTACCTGTTTTTATTTATTATTTTATACATGAATTTTATGTATCCTGGCATCTATTTATATGTCTCACCACTTGTTTCATGATATTCTCTTTTGTTGCTTTGCTTTGGAAGTATTTATTGCTCTTAATTTTCCTGGTCACTAGTTCTGAACTCTATATGACTCATTCATGTAATTAGTTTTTATTTTTGTTAAAAAAATTAAGCTTTAGATATTCTGTCACACTGGATCCCTTTACTTAGTTGTGAGGAGAATGTCTTATGTTTAGTTAAAATTTAATAACAAGTTTTATACATTGATGGTATTCTAAGAGCTGAGAATAAAAATATAATTTGTGATGAAATAAAGAAAGGGGAACAGCAAATGCACATTTCAAGACTTAAATACTTAGGAAAAAGGAAATAATCCATGTGATAATCTATAGAAATGGCATTTCATAGTGATAACATCAAATGCAAAAAGCCTAGAGGCATTATAGTTCTGAAAATTCTATGAGACCAAGTTTAGAAATTCTGGCTTCTCCTCATCGCATGGAATGGAGTCAATTGAGTTTGCCAAGGCTGTTCACTTAAAGAATACAGAATCGTCAGGATATGGATACTTTTCTAATGCATAGCAACAACAGGCTGGAACAGCCTGCACTTAGTGTGCTAAAAGAAATCCTCACAAATTAACTAAACTCAAAATATGAGTTTTCTGAGAATTTAAGCCTTCTCAGATTCTG
